# Intraoperative Assessment of Breast Cancer Tissues after Breast-Conserving Surgery Based on Mapping the Attenuation Coefficients in 3D Cross-Polarization Optical Coherence Tomography

**DOI:** 10.3390/cancers15092663

**Published:** 2023-05-08

**Authors:** Ekaterina Gubarkova, Elena Kiseleva, Alexander Moiseev, Dmitry Vorontsov, Sergey Kuznetsov, Anton Plekhanov, Maria Karabut, Marina Sirotkina, Grigory Gelikonov, Sergey Gamayunov, Alexey Vorontsov, Petr Krivorotko, Natalia Gladkova

**Affiliations:** 1 Institute of Experimental Oncology and Biomedical Technologies, Privolzhsky Research Medical University, 10/1 Minin and Pozharsky Sq., 603950 Nizhny Novgorod, Russia; kiseleva84@gmail.com (E.K.); strike_gor@mail.ru (A.P.); maria.karabut@gmail.com (M.K.); sirotkina_m@mail.ru (M.S.); natalia.gladkova@gmail.com (N.G.); 2 Institute of Applied Physics of the Russian Academy of Sciences, 46 Ulyanova St., 603950 Nizhny Novgorod, Russia; aleksandr.moiseev@gmail.com (A.M.); grig@ufp.appl.sci-nnov.ru (G.G.); 3 Nizhny Novgorod Regional Oncologic Hospital, 11/1 Delovaya St., 603126 Nizhny Novgorod, Russia; dr.vorontsovdmitriy@rambler.ru (D.V.); zunek@mail.ru (S.K.); gamajnovs@mail.ru (S.G.); doctorvorontsov@mail.ru (A.V.); 4 N.N. Petrov National Medicine Research Center of Oncology, 68 Leningradskaya St., 197758 St. Petersburg, Russia; dr.krivorotko@mail.ru

**Keywords:** cross-polarization optical coherence tomography (CP-OCT), attenuation coefficient, breast cancer subtypes, tumorous and non-tumorous breast tissue, breast-conserving surgery (BCS)

## Abstract

**Simple Summary:**

Multiple technological solutions are being explored to be used in the intraoperative assessment of resection margins in breast cancer and detection of the residual tumor cells during breast-conserving surgery (BCS) for the purpose of reducing the need for a re-resection. We applied the cross-polarization optical coherence tomography (CP OCT) method for intraoperative ex vivo human breast cancer specimens imaging and performed a qualitative and quantitative assessment of 3D CP OCT data using a depth-resolved approach of measuring the attenuation coefficient estimation in co- and cross-polarization channels. *En face* color-coded attenuation coefficient maps were constructed, and targeted calculations of the characteristic median value of the attenuation coefficients in both channels were performed for different breast tissue regions. As a result, highly accurate differentiation of the tumorous from non-tumorous breast tissue was achieved. This new optical technique with estimation attenuation coefficients of volumetric CP OCT data can be used as an innovative adjunct intraoperative tool to evaluate resection margins during BCS and to perform a targeted histological biopsy.

**Abstract:**

Intraoperative differentiation of tumorous from non-tumorous tissue can help in the assessment of resection margins in breast cancer and its response to therapy and, potentially, reduce the incidence of tumor recurrence. In this study, the calculation of the attenuation coefficient and its color-coded 2D distribution was performed for different breast cancer subtypes using spectral-domain CP OCT. A total of 68 freshly excised human breast specimens containing tumorous and surrounding non-tumorous tissues after BCS was studied. Immediately after obtaining structural 3D CP OCT images, *en face* color-coded attenuation coefficient maps were built in co-(Att(co)) and cross-(Att(cross)) polarization channels using a depth-resolved approach to calculating the values in each A-scan. We determined spatially localized signal attenuation in both channels and reported ranges of attenuation coefficients to five selected breast tissue regions (adipose tissue, non-tumorous fibrous connective tissue, hyalinized tumor stroma, low-density tumor cells in the fibrotic tumor stroma and high-density clusters of tumor cells). The Att(cross) coefficient exhibited a stronger gain contrast of studied tissues compared to the Att(co) coefficient (i.e., conventional attenuation coefficient) and, therefore, allowed improved differentiation of all breast tissue types. It has been shown that color-coded attenuation coefficient maps may be used to detect inter- and intra-tumor heterogeneity of various breast cancer subtypes as well as to assess the effectiveness of therapy. For the first time, the optimal threshold values of the attenuation coefficients to differentiate tumorous from non-tumorous breast tissues were determined. Diagnostic testing values for Att(cross) coefficient were higher for differentiation of tumor cell areas and tumor stroma from non-tumorous fibrous connective tissue: diagnostic accuracy was 91–99%, sensitivity—96–98%, and specificity—87–99%. Att(co) coefficient is more suitable for the differentiation of tumor cell areas from adipose tissue: diagnostic accuracy was 83%, sensitivity—84%, and specificity—84%. Therefore, the present study provides a new diagnostic approach to the differentiation of breast cancer tissue types based on the assessment of the attenuation coefficient from real-time CP OCT data and has the potential to be used for further rapid and accurate intraoperative assessment of the resection margins during BCS.

## 1. Introduction

Breast cancer remains the most commonly diagnosed cancer (around 30% of all newly diagnosed cancers each year) among women worldwide [1,2]. Currently, advances in multimodal imaging allow its early detection. The most recommended treatment for early-stage breast cancer is breast-conserving surgery (BCS); during the process, the surgeon needs to assess the extent of the disease accurately and its margin status to reduce the likelihood of local recurrence and the need for a re-resection [3,4]. The main criterion for detection and confirmation of optimal BCS intraoperatively is the negative resection margins around the primary tumor (the margin is defined as the distance from the tumor to the cut surface of the resection specimen) [5,6,7]. Difficulties in making the diagnostic decision on where to draw the resection lines occur in patients who have been treated with neoadjuvant chemotherapy due to the possible development of a scattered response in some types of breast cancer. Several factors need to be accounted for in the evaluation of the resection margin, such as the heterogeneity of breast cancer, the prognosis of the disease course (which is dependent on the degree of malignancy or aggressiveness), and the choice of treatment tactics [8,9,10]. Additionally, the margins of the breast specimen may not be clear after primary surgery when invasive breast cancer is accompanied by ductal carcinoma in situ (DCIS) and/or lobular breast cancer. Therefore, there exists a significant clinical need for a better intraoperative margin assessment and targeted surgery in combination with targeted therapy which involves the use of cytotoxic agents, immunotherapy, and intraoperative radiation therapy [11].

At present, several methods of intraoperative margin assessment are part of standard care, but they all have significant clinical and technical limitations. Intraoperative tumor margin evaluation can be performed using frozen section analysis and imprint cytology [12]. However, these techniques have several limitations, such as resource intensity, technical difficulties in preparing adipose tissue, sampling only a small percentage of the surgical margins, and limited efficacy, especially for DCIS. Thus, there has been increased research interest in deploying new intraoperative high-resolution technologies for the delineation of tumor morphology and the precise estimation of breast cancer size and localization in real-time with the aim of achieving clear resection margins and therefore reducing the BCS re-excision rate [13]. The most promising techniques for intraoperative margin assessment are various optical methods due to their rapid acquisition of image data, label-free imaging technique, and penetration depths sufficient to meet consensus guidelines for establishing clear margins in BCS. These methods include handheld probe-based radiofrequency spectral analysis [14], quantitative diffuse reflectance imaging [15], confocal mosaicking microscopy [16], point spectroscopy [17], and optical coherence tomography (OCT) [18,19,20,21].

OCT is the most promising method for intraoperative assessment of breast cancer due to its high resolution (5–15 μm) and label-free imaging modality that yields real-time 3D images of tissue microstructure at high speed to depths of up to 2 mm in biological tissues. Furthermore, OCT can be miniaturized into a handheld or needle probe [22,23] to enable local diagnosis and assessment of resection margins with micrometer-resolution for targeted breast surgery. In earlier studies, traditional OCT was introduced as a high-resolution imaging tool for differentiating between malignant tumors and fibro-adipose tissues [18,21,24]. In addition, OCT has been used for intraoperative assessment of resection margins during BCS [19,20,21,22]. At the same time, on conventional structural log-scale OCT images, dense malignant tissue, and normal connective tissue can be poorly differentiated due to their similar refraction and high scattering intensity. The interaction between parenchyma and stroma in invasive breast cancer determines its aggressive biological behavior, resistance to chemotherapeutic treatment, and various survival rates depending on the degree of the tumor malignancy [25]. Tumor stroma state assessment is fundamentally important because the collagen matrix plays a crucial role in breast cancer invasion and metastatic spreading [26,27]. It is necessary to search for new OCT criteria to increase the information content provided by differences in the optical properties of tissues in the diagnosis of breast cancer. These criteria may include quantification of tissue stiffness based on OCT-elastography [28,29], calculation of the attenuation coefficient [30], and birefringence contrast (specifically, differences in the collagen content) between non-tumorous and tumorous tissues based on polarization-sensitive (PS) OCT for intraoperative breast margin assessment [31,32]. Recently, there has been increasing interest in the automated quantitative characterization of human breast tissue types for surgical margin assessment based on machine learning segmentation of OCT [33,34] and PS OCT images [35,36]. It allows for identifying optimal threshold values of the optical and polarization criteria for classifying malignant tumors, fibro-adipose, and stromal tissue among human breast tissues.

Cross-polarization (CP) OCT is a variant of PS OCT that allows imaging of the initial polarization state changes due to both birefringence and cross-scattering in biological tissues which makes it possible to assess different states of the connective tissue [37]. Only orthogonally polarized backscattered light, which is mutually coherent with the incident wave, contributes to the cross-polarization image. A number of studies have shown that CP OCT is a promising method for differentiating tumorous from non-tumorous breast tissue [29,38] and human brain tissue [39], as well as for the diagnosis of bladder cancer [40]. CP OCT can also measure the attenuation coefficient, which can be helpful for improving and increasing the contrast between tumorous and non-tumorous breast tissues [41,42,43]. Various morphological features of breast cancer are anticipated to influence the polarization qualities of tumor tissue differently. This stimulates interest in the evaluation of the clinical potential of CP OCT techniques for breast cancer subtypes (malignancy grade) determination, detection of tumor borders, and improving the reliability of the negative resection margin assessment during BCS.

Despite technological development over the past decade, there are several challenges in performing high-level breast cancer tissue characterization using OCT data. They are caused by a wide range of morphological features exhibited by breast tissues under OCT examination, and a large number of individual 2D images are generated from volumetric OCT data requiring assessment. Therefore, there exists a need for new approaches to allow the quantitative analysis of OCT data and its automatization. In this study, we present for the first time attenuation coefficient maps in co- and cross-polarization channels in order to differentiate molecular breast cancer subtypes between each other and non-tumorous tissue. Furthermore, the determination of the thresholding of the attenuation coefficient values and their diagnostic significance in differentiating breast tissue types and detecting clusters of tumor cells was performed as the next step toward introducing OCT technology into the BCS protocol.

The main aims of this study are: (1) to apply mapping of the attenuation coefficients in 3D CP OCT for intraoperative differentiation of breast cancer tissues and resection margins assessment during BCS; (2) to determine the diagnostic accuracy of attenuation coefficients in co- and cross-polarization channels for delineation of non-tumorous and tumorous breast tissues.

## 2. Materials and Methods

### 2.1. Human Breast Specimens after BCS 

**Figure 1:**
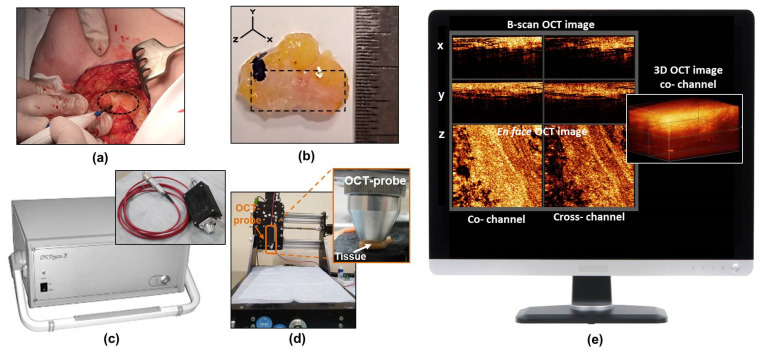
The procedure for intraoperative breast cancer assessment using CP OCT. (**a**) isolation of the breast cancer for the subsequent study; (**b**) the photo of a typical breast-tissue specimen; (**c**) CP OCT setup with the OCT probe; (**d**) special motorized table for convenient positioning of the OCT probe above the specimen and bringing it into contact with the tissue surface during scanning; (**e**) an example of 3D OCT data set on the computer monitor acquired from real-time tissue scanning. The dotted oval in panel (**a**) shows the region of the tumor to be resected in the patient’s breast tissue, and a dashed rectangle in panel (**b**) shows the area from which CP OCT images were obtained.

The study was carried out on freshly excised human specimens of breast lesions obtained from 68 patients (age 30–76) with stage I or II (T1–2 N0–1 M0) breast cancer undergoing BCS (Figure 1a). Before BCS, five patients received neoadjuvant (preoperative) chemotherapy with the combined use of doxorubicin, cyclophosphamide, and paclitaxel according to the clinical guidelines (RUSSCO Clinical Practice Guidelines [44], corresponding to generally accepted guidelines [45]). All specimens contained tumorous and non-tumorous breast tissue. The size of the specimens varied from 0.5 × 1.0 × 0.5 cm to 1.0 × 2.0 × 0.5 cm (Figure 1b).

The study was approved by the Institutional Review Board of the Privolzhsky Research Medical University and the Nizhny Novgorod Oncology Clinic (Protocol #10 from 28 September 2018 and Protocol #12 from 23 December 2021, Nizhny Novgorod, Russia). Informed consent was obtained from all participants enrolled in the study and/or their next of kin.

### 2.2. CP OCT Setup and Data Acquisition

A spectral domain CP OCT setup (Institute of Applied Physics of the Russian Academy of Sciences, Nizhny Novgorod, Russia) was used in the study for intraoperative visualization of breast cancer tissue (Figure 1c) [37]. The setup combines traditional structural OCT and polarization modes. As a result, two images are recorded simultaneously: an image in co-polarization (reflected light with a polarization state parallel to the initial polarization state) and an image in cross-polarization (reflected light with changed polarization which is orthogonal to the initial one) channels (Figure 1e). The CP OCT setup has a common-path interferometric layout that operates at a 1.3 μm central wavelength with an axial resolution of ~10 μm and a lateral resolution of ~15 μm. It has a 20 kA-scans/sec scanning rate and performs 2D lateral scanning with a range of 256 × 256 B-scans to obtain a 3D distribution of backscattering light in two polarization states. The scanned volume of 2.4 × 2.4 × 1.5 mm was acquired over 26 s. A special motorized table was used to conveniently position the OCT probe above the specimen and bring it into contact with the tissue (Figure 1d).

The 3D CP OCT images of the fresh breast tissue were acquired immediately after tumor resection (Figure 1e). The distribution of the optical attenuation coefficients was generated in real-time during the acquisition process. Each specimen was used to acquire 10–14 3D CP-OCT images (in 1–2 rows) depending on the size of the specimen and were taken with the image overlap to attain data from the entire surface being studied. The total scanning time along a 1–2 cm trajectory on a specimen was 3–5 minutes, depending on the number of stitched images. In total, greater than 600 3D CP OCT images with corresponding attenuation coefficient maps of breast tissue were obtained.

### 2.3. CP OCT Data Processing

Quantitative assessment of the 3D CP OCT images of breast tissue (characterized by high optical and morphological heterogeneity) was based on calculating two optical coefficients using a depth-resolved approach: the commonly used rate of attenuation in the co-(Att(co)) and cross-(Att(cross)) polarization channels. We expect that the additional usage of Att(cross) may provide us with more information about the morphological features of breast cancer tissue. The results were shown as *en face* color-coded maps to present the 2D distribution of these coefficients for different breast tissue types.

The depth-resolved approach was applied for the quantitative assessment of OCT data in co-polarization. Such an approach was proposed in [46] under the assumption that the backscattering coefficient is proportional to the attenuation coefficient with the constant ratio between the two in the OCT depth range. As shown in [46], the attenuation coefficient could be estimated as follows:

(1) $$\mu_{i}^{att}=\frac{I_{i}}{2\Delta \sum_{i+1}^{i_{\max}}I_{j}}$$

where *I_i_* is the noise-free OCT signal amplitude in the co- or cross-channel, *µ_i_^att^* is the corresponding specimen attenuation coefficient estimation, *i*—is the axial measurement number, *i*_max_—is the total number of pixels in the axial direction, and Δ is the pixel size along the axial dimension.

Due to the presence of the non-zero additive noise in the experimental OCT signal amplitude distribution, the direct application of Equation (1) to the measured OCT signal will lead to the following estimation:

(2) $$\mu_{i}^{est}=\frac{I_{i}+N_{i}}{2\Delta \sum_{i+1}^{i_{\max}}\left(I_{j}+N_{j}\right)}$$

where *N_i_* is the additive noise, *µ_i_^est^* is the attenuation coefficient estimation obtained from OCT data in the presence of noise. To mitigate the effect of the noise, in [42], it was proposed to reorganize Equation (2) as follows to improve the estimation:

(3) $$\begin{array}{l} \mu_{i}^{est}=H_{i}\cdot \mu_{i}^{att}+N_{i}^{\mu },\\ \mathrm{where}\\ H_{i}=1-\frac{\sum_{i+1}^{\infty }N_{j}}{\sum_{i+1}^{\infty }I_{j}+\sum_{i+1}^{i_{\max}}N_{j}}=1-\frac{\langle N\rangle \cdot (i_{\max}-i)}{\sum_{i+1}^{\infty }I_{j}+\sum_{i+1}^{i_{\max}}N_{j}}\\ N_{i}^{\mu }=\frac{N_{j}}{2\Delta \sum_{i+1}^{i_{\max}}(I_{j}+N_{j})}=\frac{\langle N\rangle }{2\Delta \sum_{i+1}^{i_{\max}}(I_{j}+N_{j})} \end{array}$$

where 〈*N*〉 is the mean noise amplitude.

Such reorganization of the equation allows one to obtain the estimation of the attenuation coefficient according to Equation (1) from the estimation according to Equation (2):

(4) $$\mu_{i}^{att}=\frac{H_{i}\cdot SNR_{i}^{\mu }}{{\left|H_{i}\right|}^{2}\cdot SNR_{i}^{\mu }+1}\cdot \mu_{i}^{est}$$

where $SNR^{\mu }{}_{i}$ is the local signal-to-noise ratio (*SNR*).

One should note that according to [42], the depth-dependent sensitivity of the OCT system due to confocality and spectral roll-off will lead to the attenuation coefficient estimation bias, which will not exceed 10%; thus, these factors were not considered in the present study. One should also note that to reduce the effect of the speckle noise on the local attenuation coefficient estimation, the volume of measured OCT data was averaged in the local 3 × 3 × 3 pixel window before the attenuation coefficient estimations.

**Figure 2:**
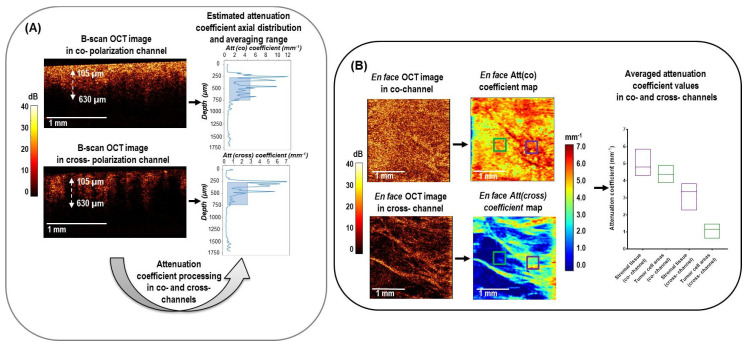
Design of 3D CP OCT image processing from cross−sectional log−scale OCT images to the *en face* attenuation coefficient mapping and statistics distribution in studied tissues. (**A**) cross−sectional OCT images in co− and cross− polarization channels were processed according to Equation (4) to obtain the depth−resolved attenuation coefficient distributions. The estimated attenuation coefficient axial distribution was then averaged in a depth range of 105−630 µm from the specimen surface (blue box); (**B**) This plot explains that after calculation of averaged attenuation coefficient value in each A−scan, *en face* color−coded attenuation (Att) coefficients maps are built for each of the two polarization channels. These maps are compared with the structural *en face* CP OCT images and serve to select regions of interest for calculating the local attenuation coefficient of a particular tissue (green and purple squares) for subsequent statistical analysis.

Figure 2 shows the outline of the data processing used to calculate both attenuation coefficients in co- and cross-polarization channels from the OCT signal. Figure 2A shows examples of B-scans in co- and cross-polarization channels for breast tissue with corresponding random A-scans, demonstrating different speeds of attenuation of the signal with depth, which was calculated using an adaptive depth-resolved approach to the attenuation coefficient calculation. The estimated volume distributions of the optical coefficients were shown as 2D *en face* color-coded maps with averaging in the predefined depth range (Figure 2B). For each collected A-scan set, both attenuation coefficients were calculated in the same predefined depth range starting from ∼105 μm below the tissue surface to a depth of ∼630 μm (Figure 2A). The choice of depths was determined by the construction of the most contrasting color-coded maps in this range, providing the best information about the morphology of breast tissue. The 2D *en face* color-coded maps were constructed based on the distribution of coefficient values for each OCT image in co- and cross-polarizations (Figure 2B). Color-coded maps provide information about the tissue properties in the range of specified depths; therefore, they are easier to interpret, and they visualize the tumor in a more contrasted way than the original *en face* log-scale structural OCT images, for the analysis of which only one plane from the tissue surface is selected. The color settings of each map reflect the values of the corresponding attenuation coefficient, where areas with a high signal decay rate are represented by shades of yellow and red colors and areas with low attenuation by shades of blue and light blue.

### 2.4. Histological Study

After CP OCT imaging of the breast tissue, the scanning area was marked with histological ink for correlation with histology. *En face* histological sections were prepared through the marked areas so that their planes corresponded to the *en face* CP OCT and *en face* attenuation coefficient images. Multiple 7µm thick serial sections were taken from a single specimen block, with 35 µm discarded between levels and each section stained with hematoxylin and eosin (H&E) to determine the breast cancer type and additional sections stained with Van-Gieson’s solution in order to assess collagen content. Histological samples were examined using the EVOS M7000 Imaging System (Thermo Fisher Scientific Inc., Waltham, MA, USA) in transmitted light. Histopathological analysis was performed by a single experienced pathologist (S.K.).

The identified histological types of breast lesions include fibro-adenomatosis (*n* = 3), fibroadenoma (*n* = 3), micro-invasive carcinoma with ductal carcinoma in situ (DCIS) (*n* = 3), invasive lobular carcinoma (ILC) (*n* = 18), invasive carcinoma of no special type (NST) (*n* = 36) with high-grade or low-grade cancer cells and breast specimens after neoadjuvant chemotherapy with a partial pathological response (*n* = 5). All 68 breast lesions studied contained both tumor and peri-tumoral non-tumorous tissues (adipose and fibrous connective tissue). Special attention was paid to the tumor stroma of the mammary gland, which has different dystrophic changes in collagen fibers (fibrosis or hyalinosis). In addition, different molecular subtypes of breast cancer are characterized by a special structure of the connective tissue and its certain proportion with tumor (malignant) cells.

Accurate disease prognosis and optimization of individual therapy options require differentiation of molecular breast cancer subtypes. Therefore, an immunohistochemistry assessment, including the analysis of estrogen receptor (ER), progesterone receptor (PR), Her2/neu, and antigen Ki-67 expression, was carried out. All invasive and micro-invasive breast cancer cases (*n* = 62) were divided into five molecular subtypes: Luminal A (ER+, PR+, Her2/neu-, Ki-67 < 15) (*n* = 14), Luminal B (EP+, PR+, Her2/neu-, Ki-67 > 15) (*n* = 25), Luminal B (EP+, PR+, Her2/neu+, Ki-67 > 15) (*n* = 15), non-luminal (ER-, PR-, Her2/neu+, Ki-67>15) (*n* = 5) and triple-negative (ER-, PR-, Her2/neu-, Ki-67 > 15) (*n* = 3).

To assess the tumor response to therapy, Residual Cancer Burden (RCB) histological grading system was used. RCB is an integral criterion widely used in clinical practice that allows the prediction of relapse-free survival based on the size of the residual breast tumor, cellularity, and the number and size of the affected lymph nodes [47].

### 2.5. Correlation of the CP OCT Data with Histology and Region of Interest Selection

The results of histopathology were compared with the corresponding CP OCT-based findings. After saving *en face* log-scale structural CP OCT images acquired at a depth of ∼150 μm from the tissue surface and constructing *en face* color-coded attenuation coefficient maps at a depth range of 105–630 µm, we selected one of the *en face* histological sections prepared from the same sample from a depth of ~150–200 µm that best matched the attenuation coefficient maps. Despite the efforts to select the area of comparison as accurately as possible, complete correspondence between the *en face* CP OCT and *en face* attenuation coefficient maps on the one hand and the corresponding *en face* histological images, on the other hand, could not be achieved. We identified several reasons for this. First, the shape of the sample could be distorted or deformed during the histological preparation procedures due to the fixation in formalin, dehydration, paraffining, etc. [48]. Second, a slight difference in the histological slice positions (even ~tens of microns) also affects the geometry of the breast tissue structural components and complicates the comparison with the OCT-based images of the fresh tissue. Third, although OCT images have a micrometer-scale resolution (typically 10–15 μm) approaching that of one of histology [49], they do not typically reach the cellular level of histology; therefore, slight discrepancies in the size of individual structural components of the tissue are possible [50]. Given the above, only larger (~tens of microns or more) regions or components can be matched at this scale, while smaller structures (~several microns) may appear much more distorted and displaced.

Therefore, in this study, to provide more accurate identification of certain breast tissue structures on color-coded attenuation coefficient maps, we scanned a large sample field and then analyzed the area consisting of 10–14 stitched *en face* CP OCT images and the corresponding attenuation coefficient maps. On such stitched *en face* OCT-based images, we analyzed the topological similarity of the mutual positions and geometrical sizes of the structural components of tumorous and non-tumorous breast tissue and the corresponding *en face* histology sections.

For a comparative numerical analysis of data in each color-coded attenuation coefficient map, corresponding histological regions of interest (ROIs) were selected for calculating the local attenuation coefficient. In doing so, we first selected ROIs based on the log-scale structural CP OCT images and then assessed the attenuation coefficient maps. A total of 165 ROIs for different breast tissue types in each attenuation coefficient map were selected for quantitative statistical analysis and were categorized into five types: adipose tissue (*n* = 33), the non-tumorous fibrous connective tissue of breast (*n* = 36), hyalinized tumor stroma (*n* = 27), fibrotic tumor stroma with low-density tumor cells (*n* = 34), high-density tumor cells (*n* = 35) (*n*—number of images). The typical size of each ROI was 300 × 300 µm, over which the median values of the attenuation coefficients were calculated, as shown in the color-coded maps by the colored squares (Figure 2B). For a fairly limited number of the studied breast tissue images (*n* = 165), the ROI-windows on the color-coded attenuation coefficient maps were selected manually by exact matching with the histology. The manual ROI selection was used with the understanding that if a significant difference between these areas were observed, the next research stage would be moving towards more automated processing to develop an automatic procedure of breast tissue differentiation in the future.

### 2.6. Statistical Analysis

The variables for statistical inter-group comparison were the Att(co) and Att(cross) coefficients calculated from 3D CP OCT images. To evaluate the results of quantitative image processing, we used the mean and median values among all values of every optical coefficient calculated for each A-scan of a 3D CP OCT image. The results are expressed as Me [Q1; Q3], where Me is the median of the analyzed parameter and [Q1; Q3] are the 25th and 75th percentile values, respectively. Since this study includes a comparison of multiple groups, the Mann–Whitney U-test with Bonferroni correction was selected. In all cases, the differences were considered statistically significant when *p* < 0.05.

The assessment of the informative value and diagnostic capabilities of the studied method was carried out with an estimation of sensitivity (Se), specificity (Sp), and diagnostic accuracy (Ac). Based on the Se and Sp values, the receiver operating characteristic (ROC) curves were constructed, which show the dependence of the number of a true positive rate on the number of a false positive rate. For quantitative characterization of the ROC curves, we evaluated the area under the ROC curve (AUC), i.e., the area bounded by the ROC curve and the axis of the false positive rate [51]. The higher the AUC is, the better the classifier.

For statistical data processing, the program Statistical Package for Social Sciences 16.0 (SPSS, Chicago, IL, USA) was used. The ROC-related calculations of the sensitivity, specificity, diagnostic accuracy, and area under the ROC were performed with Prism 8.0.2 statistical software (GraphPad Software, San Diego, CA, USA).

## 3. Results

### 3.1. Color-Coded Attenuation Coefficient Maps in Differentiation of Breast Cancer Subtypes

In the first part of this study, stitched *en face* color-coded attenuation coefficient maps in co-(Att(co)) and cross-(Att(cross)) polarization channels from benign (non-cancerous) fibroadenoma, four cases of invasive breast cancer with different grades of malignancy (having different molecular subtypes) and a case of invasive breast cancer after neoadjuvant chemotherapy were analyzed. First, we conducted their visual assessment vis-a-vis histological images and distinguished a characteristic pattern with the predominant color palette of the attenuation coefficient maps. Second, we discussed inter- and intra-tumor heterogeneity of various breast cancer subtypes and the features of their boundaries and also noted the advantages of the attenuation coefficient imaging compared to *en face* log-scale structural CP OCT images.

**Figure 3:**
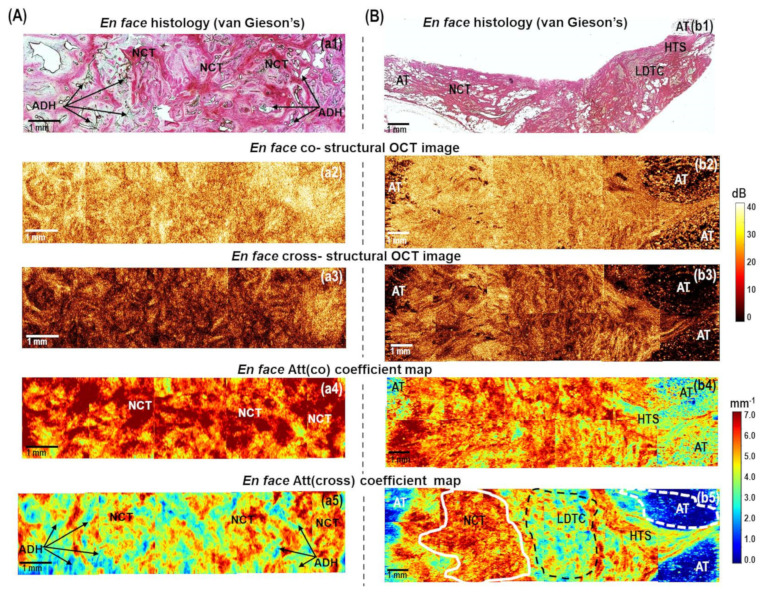
The representative cases of (**A**) benign breast fibroadenoma and (**B**) low−grade invasive carcinoma of no special type (Luminal A molecular subtype): CP OCT imaging with attenuation coefficient mapping. *En face* histology (**a1**,**b1**) corresponding to the stitched *en face* log−scale structural CP OCT images (**a2**,**a3**,**b2**,**b3**) acquired at a depth of ∼150 μm from the tissue surface and the stitched *en face* color−coded attenuation coefficient maps (**a4**,**a5**,**b4**,**b5**) constructed at a depth range of 105−630 µm. Images (**a2**,**a4**,**b2**,**b4**) are in co− and (**a3**,**a5**,**b3**,**b5**) cross− polarization channels. Non−tumorous fibrous connective tissue has high Att(co) and Att(cross) attenuation coefficient values (from yellow to red colors, indicated with a white line in (**b5**)); regions of atypical ductal structures have decreased values of attenuation coefficients (light blue and dark blue colors, arrows in (**a5**)); low−density tumor cells in the fibrotic tumor stroma have medium values of Att(cross) coefficient (from light blue to yellow colors, black dotted line in (**b5**)); adipose tissue have the lowest values of Att(cross) coefficients (white dotted line in (**b5**)). Abbreviations: Att—attenuation, AT—adipose tissue, NCT—non-tumorous fibrous connective tissue, ADH—atypical ductal hyperplasia, LDTC—low−density tumor cells in the fibrotic tumor stroma, HTS—hyalinized tumor stroma.

Figure 3A shows the case of benign breast condition (fibroadenoma) containing both glandular tissue (ducts and lobules) and non-tumorous fibrous connective tissue. Histologically it appears quite heterogeneous: most of the fibroadenoma is characterized by the growth of fibrous connective tissue around the atypical glandular structures of the mammary gland, squeezing of the ducts, which take the form of narrow slits, and atypical ductal hyperplasia. (Figure 3(a1)). These structures have opposite scattering properties: the fibrous connective tissue enhances the OCT signal due to densely packed collagen bundles; the ducts and lobules, on the other hand, transmit light, and the corresponding areas in *en face* CP OCT images appear dark (with a greatly reduced signal level or its absence) (Figure 3(a2,a3)). Narrowed lumen of the ducts due to fibrosis and reduced lobules are too small to be detected in log-scale CP OCT images, but if they are distinguished among fibrous connective tissue, they look more contrasted in cross-polarization images (Figure 3(a3)). The *en face* color-coded attenuation coefficient maps (Figure 3(a4,a5)) seem to be more contrasted in the visualization of heterogeneity of fibroadenoma compared to log-scale CP OCT images (Figure 3(a2,a3)). For dense fibrous connective tissue, higher attenuation coefficient values are typical: in Att(co) coefficient maps more than 5.0 mm^−1^ (Figure 3(a4), red and orange colors), in Att(cross) coefficient maps more than 5.0 mm^−1^ (Figure 3(a5), red and yellow colors); regions of the ductal and lobular structures expressed with decreased values of attenuation coefficient values: in Att(co) coefficient maps less than 5.0 mm^−1^ (Figure 3(a4), yellow color), in Att(cross) coefficient maps less than 3.0 mm^−1^ (Figure 3(a5), bright blue and dark blue colors). Att(cross) coefficient maps compared to Att(co) coefficient maps distinguish non-tumorous fibrous connective tissue from ducts/lobules structures in a more contrasted way.

**Figure 4:**
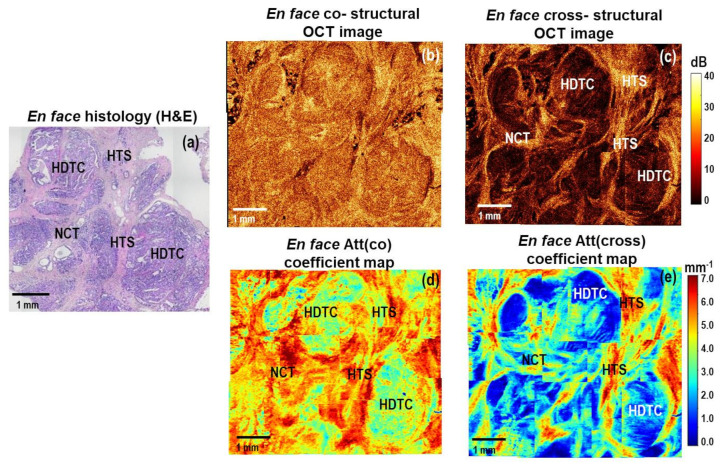
A representative case of high−grade micro−invasive ductal carcinoma and ductal carcinoma in situ (DCIS): CP OCT imaging with attenuation coefficient mapping. *En face* histology (**a**) corresponding to the stitched *en face* log−scale structural CP OCT images (**b**,**c**) acquired at a depth of ∼150 μm from the tissue surface and the stitched *en face* color−coded attenuation coefficient maps (**d**,**e**) constructed at a depth range of 105−630 µm. Images (**b**,**d**) are in co− and (**c**,**e**) cross− polarization channels. The areas of high−density tumor cells have the lowest values of Att(cross) coefficient (dark blue colors); the areas of hyalinized tumor stroma have the highest values of Att(cross) coefficient (red color); the areas of non-tumorous fibrous connective tissue have slightly lower values of Att(cross) coefficient (from yellow to red colors). Abbreviations: Att—attenuation, HDTC—high−density tumor cells in duct (DCIS), NCT—non−tumorous fibrous connective tissue, HTS—hyalinized tumor stroma.

**Figure 5:**
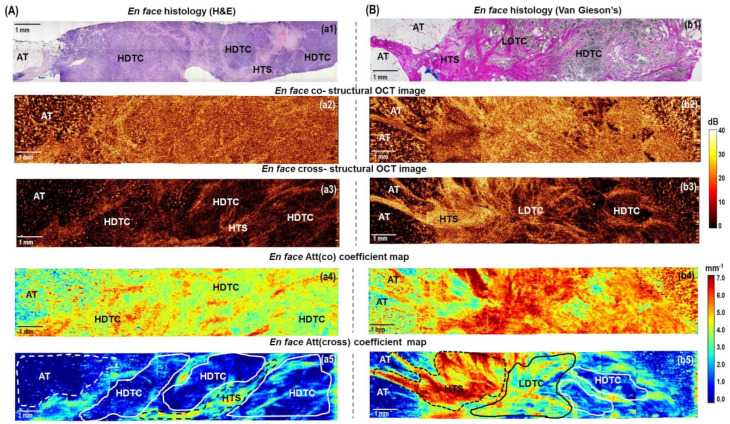
The representative cases of high−grade invasive (**A**) ductal carcinoma (Luminal B(Her2Neo−) subtype) and (**B**) lobular carcinoma (Luminal B (Her2Neo−) subtype): CP OCT imaging with attenuation coefficient mapping. *En face* histology (**a1**,**b1**) corresponding to the stitched *en face* log−scale structural CP OCT images (**a2**,**a3**,**b2**,**b3**) acquired at a depth of ∼150 μm from the tissue surface and the stitched *en face* color−coded attenuation coefficient maps (**a4**,**a5**,**b4**,**b5**) constructed at a depth range of 105−630 µm. Images (**a2**,**a4**,**b2**,**b4**) are in co− and (**a3**,**a5**,**b3**,**b5**) cross− polarization channels. The areas of high-density tumor cells (white lines in (**a5**) and (**b5**)) and adipose tissue have the lowest Att(cross) coefficient values (white dotted line in (**a5**) and (**b5**)); the area of low−density tumor cells in the fibrous tumor stroma have the medium Att(cross) coefficient values (black line in (**b5**)); the areas of hyalinized tumor stroma have the highest values of Att(cross) coefficient (yellow and red colors, black dotted line in (**a5**) and (**b5**)). Abbreviations: Att—attenuation, AT—adipose tissue, NCT—non−tumorous fibrous connective tissue, LDTC—low−density tumor cells, HDTC—high−density tumor cells, HTS—hyalinized tumor stroma.

Figure 3B, Figure 4, and Figure 5 demonstrate several examples of the invasive breast cancer subtypes, which differ in the quantitative ratio of the connective tissue and tumor cell clusters, the localization of these clusters, and the different tumor cell density. The presented examples also contain boundaries with non-tumorous adipose and fibrous connective tissue, which allow for the evaluation of the quality of OCT visualization of tumor margins using the calculation of attenuation coefficients and without them.

**Figure 6:**
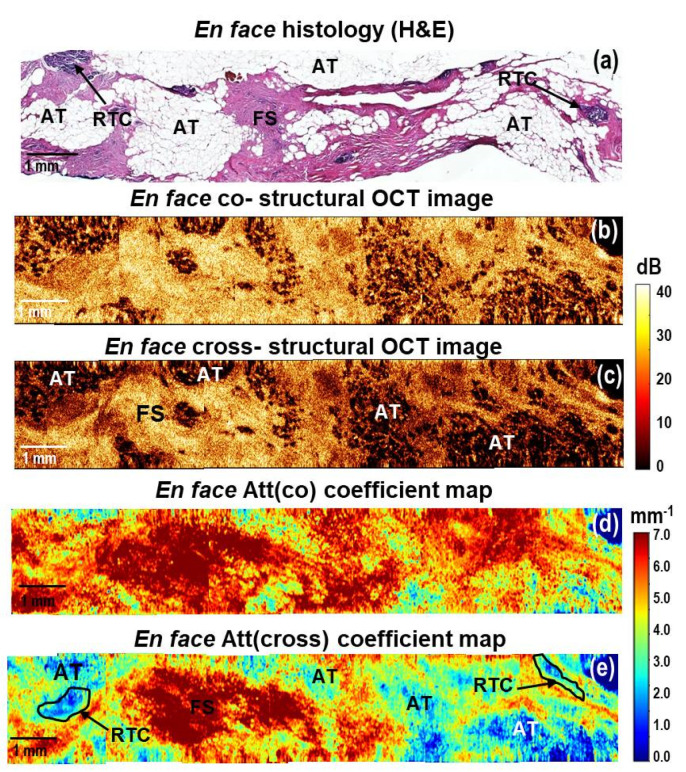
A representative case of breast cancer specimens (Luminal B (Her2Neo+) subtype) after courses of neoadjuvant chemotherapy: CP OCT imaging with attenuation coefficient mapping. *En face* histology (**a**) corresponding to the stitched *en face* log−scale structural CP OCT images (**b**,**c**) acquired at a depth of ∼150 μm from the tissue surface and the stitched *en face* color−coded attenuation coefficient maps (**d**,**e**) constructed at a depth range of 105–630 µm. Images (**b**,**d**) are in co− and (**c**,**e**) cross− polarization channels. Att (cross) coefficient map detects better residual tumor cells, which are characterized by the lowest coefficient values (black line in (**e**)). Adipose tissue is better visualized in the structural OCT images compared to the Att(co) and Att(cross) coefficient maps. Abbreviations: Att—attenuation, AT—adipose tissue, RTC—residual tumor cells, FS—fibrous stroma.

To generalize, normal adipose tissue in *en face* log-scale CP OCT images in co- and cross-channels are characterized by a special cellular structure and have a very low level of the OCT signal with bright spots (Figure 3B, Figure 5, and Figure 6), tumor stroma has a considerably higher level of the OCT signal in cross-channel with commonly distinguished densely packed collagen bundles (Figure 3B, Figure 4 and Figure 5). Breast cancer tissue has a low level of the OCT signal and, as a rule, is clearly distinguished in the log-scale CP OCT images only in the presence of large foci of tumor cells (Figure 4 and Figure 5). In other cases, it is rather poorly differentiated, especially at the low density of tumor cells in the fibrotic tumor stroma (Figure 3B and Figure 5).

Figure 3B shows typical CP OCT findings for low-grade invasive breast cancer of no special type (Luminal A molecular subtype), which is the most common and less aggressive subtype with the best prognosis for treatment. Histologically, this tumor subtype has the most pronounced tumor stroma, consisting of densely intertwined collagen fibers, among which there are numerous foci of tumor cells in the form of chains or clusters of low density (Figure 3(b1)). Comparing *en face* log-scale CP OCT images (Figure 3(b2,b3)) and *en face* color-coded (Figure 3(b4,b5)) maps of this state, we can conclude that Att(cross) coefficient map demonstrates a more pronounced contrast between non-tumorous fibrous tissue and infiltrating tumor cells. Areas of decreased values of Att(cross) coefficient (less than 4.0 mm^−1^) corresponded to low-density tumor cells in the fibrotic tumor stroma (Figure 3(b5), indicated with a black dotted line), and values of high values (more than 5.0 mm^−1^) corresponded to non-tumorous fibrous connective tissue (Figure 3(b5), indicated with a white line). In this case, the border of the transition of the tumor tissue into the surrounding adipose tissue is also well visualized, which is characterized by the lowest values (less than 2.0 mm^−1^) of the Att(cross) coefficient (Figure 3(b5), indicated with a white dotted line).

It has been found that benign fibroadenoma (Figure 3A) and invasive carcinoma of a low degree of malignancy (Figure 3B) may have similar scattering and polarization properties, which also similarly affect the values of Att(co) and Att(cross) coefficients.

Figure 4 shows representative stitched *en face* log-scale CP OCT images and stitched *en face* color-coded attenuation coefficient maps for high-grade micro-invasive carcinoma and ductal carcinoma in situ (DCIS). Histologically, this case is characterized by the presence of ducts filled with tumor cells with clear boundaries, which are surrounded by fibrosis and hyalinized stroma. (Figure 4a). The presence of hyalinized stroma indicates secondary deeper (dystrophic) changes in the tumor stroma. At the same time, collagen fibers form dense homogeneous structures characterized by a prominent accumulation of proteoglycans and glycosaminoglycans [52,53]. In this case, in the log-scale OCT images in co- and cross-channels (Figure 4b,c), a fairly heterogeneous structure is visualized. Stromal tissue has a high level of OCT signal, and clusters of tumor cells with high density have a low level of OCT signal. Comparing the two color-coded attenuation coefficient maps, Att(cross) (Figure 4e) map is the most informative and fully correlates with the histological images (Figure 4a). The ducts filled with tumor cells of high density are very clearly seen, which have low values (less than 3.0 mm^−1^) of Att(cross) coefficient (Figure 4e, bright blue and dark blue colors). The areas of fibrous stroma and hyalinosis have high values of Att(cross) coefficient—4–5 mm^−1^ and more than 6 mm^−1^, consequently (Figure 4e, yellow and red colors).

Figure 5A presents a common example of CP OCT examination of high-grade invasive carcinoma of no special type (Luminal B(Her2Neo-) molecular subtype). This subtype of breast cancer, compared to the Luminal A subtype, is more aggressive and is associated with a worse treatment prognosis. It can be observed from the stitched *en face* log-scale CP OCT images that this breast cancer is characterized by a low and homogeneous level of OCT signal in the co- and cross-channels (Figure 5(a2,a3)), which is histologically confirmed by significant disorganization of the collagen fibers’ orientation and a significant increase in the tumor cell area (Figure 5(a1)). A higher concentration of tumor cells in the tumor tissue leads to a more homogeneous distribution of lower attenuation values of the Att(co) (Figure 5(a4)) and Att(cross) (Figure 5(a5)) coefficients in color-coded maps in comparison with Luminal A subtype (Figure 3B). Comparing *en face* log-scale (Figure 5(a2,a3)) and *en face* color-coded (Figure 5(a4,a5)) images of this state, we can conclude that color-coded Att(cross) map also makes it possible to identify the areas of infiltrating tumor cells with more contrast. It was shown that the areas of high accumulation of tumor cells are characterized by the lowest (less than 2 mm^−1^) values of the Att(cross) coefficient (Figure 5(a5), indicated with a white line). In addition, in this case, in the *en face* color-coded attenuation coefficient maps, the border between the tumorous and non-tumorous breast tissue was less clearly visualized by each coefficient (Figure 5(a4,a5)). This is due to the fact that high tumor cell density and adipose tissue are characterized by close low values of the coefficients (less than 4 mm^−1^). In this case, when looking for the resection margin, it is better to focus on standard log-scale CP OCT images, on which the adipose tissue has a special cellular structure, which is well distinguished from the homogeneous low level of the OCT signal in the area of tumor cells of high density (Figure 5(a2,a3)).

The cases of even more aggressive non-luminal and triple-negative breast cancers with high histologic grade and poor prognostic factors are characterized by similar scattering properties in log-scale CP OCT images, and the distribution of the Att(co) and Att(cross) coefficients was the same as for the Luminal B subtypes (Figure 5A). The homogeneous distribution of low values of attenuation coefficient areas for both breast cancer subtypes is consistent with the histologically confirmed significant disorganization of the collagen fiber orientation and a significant increase in the area of tumor cells.

Figure 5B presents an example of CP OCT findings for high-grade invasive lobular carcinoma (Luminal B (Her2Neo-) subtype), which is the second most common type of invasive breast cancer and which is characterized by an increased tendency to metastasize in comparison to Luminal A. Histologically, this type of breast cancer has a heterogeneous structure with an equal proportion of tumor cells of different density and tumorous stroma with a different density of collagen fibers (Figure 5(b1)). Stitched *en face* CP OCT images in cross-channel were also characterized by high heterogeneity of the OCT signal distribution (Figure 5(b3)), which similarly affected the Att(cross) coefficient values (Figure 5(b5)). It should be noted that in the stitched *en face* Att(co) coefficient map, it is difficult to distinguish features of the tumor structure (Figure 5(b4)). By contrast, on the stitched *en face* Att(cross) coefficient map, it is easy to distinguish the different breast tissue types and the tumor node from the surrounding adipose tissue (Figure 5(b5)). Areas of adipose tissue and high-density clusters of tumor cells were characterized by the lowest Att(cross) coefficient (less than 2 mm^−1^) values, areas of low-density tumor cells in the fibrous tumor stroma were characterized by lower Att(cross) coefficient (about 2–3 mm^−1^) values and hyalinized tumor stroma formed by densely packed collagen fibers had the highest Att(cross) coefficient (more than 6 mm^−1^) values (Figure 5(b5)). Adipose tissue is better visualized in the structural OCT images compared to Att(co) and Att(cross) coefficient maps.

Additionally, we present the results of the CP OCT study of breast cancer specimens (Luminal B (Her2Neo+) molecular subtype) from patients post neoadjuvant chemotherapy. In the case of incomplete tumor response to therapy, CP OCT could detect residual tumor cells in the tumor bed (Figure 6). In the *en face* histological images, they corresponded to the multiple foci of fibrous stroma, adipose tissue, as well as areas of single small clusters of residual tumor cells (Figure 6a). In *en face* log-scale CP OCT images, a high level of OCT signal in the areas of fibrosis and small areas with low levels of OCT signal from clusters of residual tumor cells were observed (Figure 6b,c). Fibrous stroma formed by densely packed collagen fibers is characterized by the dominance of high values (more than 6 mm^−1^) of both coefficients in the *en face* attenuation coefficient maps (Figure 6d,e), orange and red colors). The areas of residual tumor cells are contrasted only in the Att(cross) coefficient map and are characterized by the lowest (less than 2 mm^−1^) Att(cross) coefficient values (Figure 6e, indicated with a black line). At the same time, adipose tissue that was better visualized in the structural CP OCT images (Figure 6b,c) was excluded from the analysis on the color-coded attenuation coefficient map because it had similar values to tumor cell areas.

To conclude, the main result of this part of the study is that mapping the attenuation coefficients for various breast cancer subtypes significantly increases the amount of information available from the CP OCT data compared to the unprocessed log-scale CP OCT images. Tumor cell areas and fibrous connective breast tissue in log-scale structural OCT images can have similar scattering and polarization properties and, therefore, may not be contrasted. However, the calculation of local mapping of Att(cross) coefficient values makes it possible to differentiate these tissues. The calculation of the Att(cross) coefficient in comparison with Att(co) coefficient provided better contrast for the visualization of different breast tissue types (adipose tissue, non-tumorous fibrous connective tissue, hyalinized tumor stroma, high-density tumor cells and low-density tumor cells in fibrotic tumor stroma). Based on Att(cross) coefficient mapping, a considerable difference between breast cancer subtypes was revealed. This is very valuable for the exact identification of the resection boundaries of breast cancer and ensuring the cleanliness of the resection margins as well as for assessing the efficacy of therapy.

### 3.2. Comparison of Attenuation Coefficients for Breast Tissue Types Differentiation

During BCS, the main task is to detect and differentiate areas of tumor cells (regardless of their density) not only from adipose tissue but also from fibrous connective tissue to ensure the negative resection margin of the resection. Therefore, in the quantitative assessment, the non-tumorous breast tissue group was divided into the adipose and fibrous connective tissue of the mammary gland. Breast cancer tissue was divided into hyalinized tumor stroma, fibrotic tumor stroma with low-density tumor cells, and areas of tumor cells of high density.

**Figure 7:**
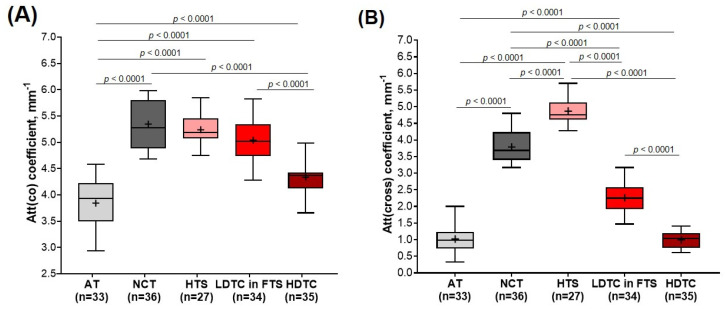
Boxplots for (**A**) Att(co−) and (**B**) Att(cross−) coefficients counted for each type of breast tissue. Centerline in the boxes —median; +—mean; box limits—25th and 75th percentiles; whiskers—minimum and maximum values. Segment indicates a statistically significant difference between the study groups (Mann−Whitney U−test with a Bonferroni correction for multiple comparisons), where *p*—is the magnitude of the statistical significance of the differences between types of breast tissue. Abbreviations: AT—adipose tissue, NCT—non-tumorous fibrous connective tissue, HTS—hyalinized tumor stroma, FTS—fibrotic tumor stroma, LDTC—low−density tumor cells, HDTC—high−density tumor cells.

Figure 7 represents diagrams comparing median values of Att(co) and Att(cross) coefficients for five breast tissue types, including adipose tissue (*n* = 33), non-tumorous fibrous connective tissue (*n* = 36), hyalinized tumor stroma (*n* = 27), low-density tumor cells in the fibrotic tumor stroma (*n* = 34) and areas of tumor cells of high density (*n* = 35). The high-density clusters of tumor cells are characterized by low values for Att(co) and Att(cross) coefficients, that is, 4.3 [4.1; 4.4] mm^−1^ and 1.0 [0.7; 1.2] mm^−1^, respectively (Figure 7A,B; dark red boxes). Tumorous tissue statistically significantly differs (*p* < 0.0001) from non-tumorous fibrous connective tissue (Att(co) = 5.3 [4.9; 5.8] mm^−1^ and Att(cross) = 3.7 [3.4; 4.2] mm^−1^) and hyalinized tumor stroma (Att(co) = 5.1 [5.0; 5.4] mm^−1^ and Att(cross) = 4.7 [4.6; 5.1] mm^−1^), as well as from adipose tissue if accounting only for Att(co) coefficient (3.9 [3.5; 4.2] mm^−1^). In the case of low-density tumor cells, when they are located as a single group or a trabecula in fibrotic tumor stroma (see Figure 3B and Figure 5B), the values of both Att(co) and Att(cross) coefficients considerably increase and constitute 5.0 [4.7; 5.3] mm^−1^ and 2.2 [1.9; 2.5] mm^−1^, respectively (Figure 7A,B; red boxes). Here tumorous tissue statistically differs from non-tumorous fibrous connective tissue, only accounting for Att(cross) coefficient (*p* < 0.0001) (Figure 7B). The calculations also demonstrate that with high statistical significance, it is possible to differentiate (*p* < 0.0001) hyalinized tumor stroma from other subtypes of breast tissue using Att(cross) coefficient (Figure 7B; light pink boxes). The increase in the Att(cross) coefficient in the hyalinized tumor stroma to 4.7 [4.6; 5.1] mm^−1^ is most likely related to the increase in the density of the arrangement of collagen fibers compared to non-tumorous connective tissue (see Figure 5B) which have values of 3.7 [3.4; 4.2] mm^−1^ (Figure 7B). Non-tumorous connective tissue is characterized by less dense and more ordered collagen fiber location, which leads to a lower signal attenuation rate in depth in the cross-channel (see Figure 3). In the case of the defibering of collagen fibers in tumor stroma and their fragmentation with an increase in the density of tumor cells, the Att(cross) coefficient values decrease significantly (see Figure 5A).

The calculations also demonstrated that adipose tissue is characterized by the lowest Att(co) and Att(cross) coefficient values and constitute 3.9 [3.5; 4.2] mm^−1^ and 0.9 [0.7; 1.2] mm^−1^, respectively (Figure 7A,B; light gray boxes). A high spread of values is associated with relatively high differences between the refractive indices of the fat cell cytoplasm and membrane. This specific structure of adipose tissue is well visualized in the log-scale OCT images (see Figure 3B and Figure 5). While there is no need to differentiate this tissue from other subtypes of breast tissue numerically, according to Att(co) coefficient values, adipose tissue statistically differs from all other four breast tissue types (*p* < 0.05).

To conclude, numerical estimation of Att(co) and Att(cross) coefficients values of different breast tissue types demonstrated that Att(cross) coefficient provides statistically significant differences for most of the analyzed tissue types, in particular for differentiation of tumor cell areas (regardless of their density) from non-tumorous fibrous connective tissue.

### 3.3. Diagnostic Accuracy of Attenuation Coefficients for Breast Tissue Type Differentiation

In the final part of this study, the diagnostic accuracy and optimal thresholds of values of both attenuation coefficients were identified to detect areas of tumor cells (regardless of their density) in the non-tumorous breast tissue. To identify optimal thresholds (Pth) of attenuation coefficients values using quantitative ROC analysis, we considered together two subtypes of tumorous tissues containing tumor cells (high-density tumor cells and low-density tumor cells in fibrotic tumor stroma) and named them tumor cell areas because, in BCS, it is important to ensure a tumor cell-free resection margin. The choice of the CP OCT diagnostic parameter (attenuation coefficients values) for the differentiation of (1) tumor cell areas from adipose tissue, (2) tumor cell areas from non-tumorous fibrous connective tissue, and (3) hyalinized tumor stroma from non-tumorous fibrous connective tissue is a trade-off between the sensitivity (Se) and specificity (Sp) rates.

**Figure 8:**
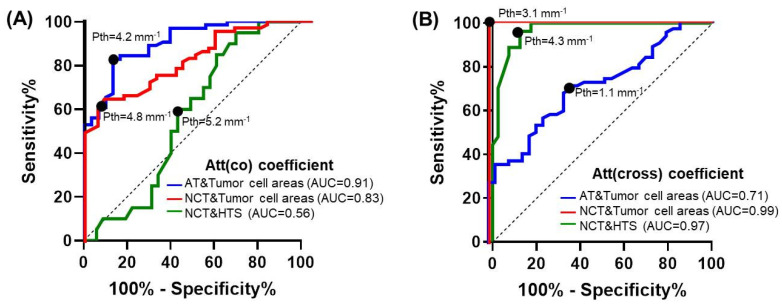
ROC−curves showing the results for distinguishing areas of tumor cells from adipose tissue (blue lines) and non−tumorous fibrous connective tissue (red lines); hyalinized tumor stroma from non−tumorous fibrous connective tissue (green lines) based on the calculation of (**A**) Att(co) and (**B**) Att(cross) coefficients. Tumor cell areas included both areas of high−density tumor cells and areas of low−density tumor cells in fibrotic tumor stroma. Black dots on the curves represent choices of the attenuation coefficients threshold value (Pth) using a trade−off between the percentages of the false negative and false positive outcomes and the greatest diagnostic accuracy. Additional abbreviations: ROC—receiver operating characteristic; AUC—area under ROC-curve; AT—adipose tissue; NCT—the non−tumorous fibrous connective tissue of the breast; HTS—hyalinized tumor stroma.

Figure 8A shows ROC curves for the detection of tumorous tissue in non-tumorous breast tissue using the attenuation coefficient in the co-channel. For differentiation between tumor cell areas and adipose tissue Att(co) coefficient threshold (Pth) equal to 4.2 mm^−1^ was proposed. This threshold is applicable at Se = 84% and Sp = 84% (Ac = 83%). The area under the ROC curve (AUC) was equal to 0.91. For differentiation between tumor cell areas and non-tumorous fibrous connective tissue Att(co) coefficient threshold (Pth) equal to 4.8 mm^−1^ was proposed. This threshold provides Se = 65% and Sp = 91% (Ac = 78%). The area under the ROC curve (AUC) was equal to 0.83. Rather low values of diagnostic accuracy indicate a clear insufficiency of this coefficient in the differentiation of these types of tissues. In addition, with this coefficient, it is practically impossible to precisely differentiate between non-tumorous fibrous connective tissue and hyalinized tumor stroma. The area under the ROC curve (AUC) was equal to 0.56.

Similarly to Figure 8A,B shows the ROC curves for the detection of tumor tissue using Att(cross) coefficient. The Att(cross) coefficient threshold value (Pth) was chosen at 1.1 mm^−1^ for the differentiation of adipose tissue from tumor cell areas, resulting in Se = 68%, Sp = 66%, and Ac = 67%. The area under the ROC curve (AUC) was equal to 0.71. The Att(cross) coefficient threshold value (Pth) was chosen equal to 3.1 mm^−1^ for the differentiation of non-tumorous fibrous connective tissue from tumor cell areas, resulting in Se = 98%, Sp = 99%, and Ac = 99%. The area under the ROC-curve (AUC) was equal to 0.99. In addition, the use of this coefficient allows good differentiation of non-tumorous fibrous connective tissue from hyalinized tumor stroma of the breast with high Se = 96%, Sp = 87%, and Ac = 91%. The area under the ROC-curve (AUC) was equal to 0.97.

Table 1 summarizes the diagnostic performance of both co- and cross-channel attenuation coefficients for the detection of different breast tissue types. It demonstrates that at the chosen thresholds of optical coefficient values, the highest diagnostic accuracy of separation of tumor cell areas and adipose tissue is 83% when calculating the Att(co) coefficient. However, in the differentiation between tumor cell areas and non-tumorous fibrous tissue, diagnostic accuracy for Att(cross) coefficient was much higher—99% against 78% for Att(co) coefficient. In addition, the Att(cross) coefficient allows better differentiation of non-tumorous fibrous tissue from hyalinized tumor stroma—diagnostic accuracy is 91% against 58% for the Att(co) coefficient.

## 4. Discussion

During BCS of invasive breast cancer, the benefits of novel imaging technologies are self-evident for intraoperative detection of any residual tumor tissue (without or after neoadjuvant treatment) in real-time. OCT appears to be a very promising tool for the routine surgical practice of an oncologist due to the advantages of this method, such as safety (a near-infrared light source is used), accuracy (micrometer-scale resolution ~ 10–15 μm), label-free imaging and high speed of obtaining 2D or 3D images of the subsurface tissue structure in real-time to a depth of 2 mm. The continuous improvements of the OCT technology in the direction of imaging speed, sensitivity, the development of functional modalities, and emerging endoscopic and handheld scanning probes, as well as OCT-signal data processing, have been the cause of the increased interest in OCT [22,23,49,54]. Furthermore, some studies apply machine learning and artificial intelligence to improve breast tissue type differentiation [33,34,35,36]. All these advances allow optimization of the analysis of large amounts of OCT data and make a visual interpretation of the OCT results more convenient for medical practitioners. Overall, the OCT method, compared to other optical methods, shows the most promise because it can collect information in a short time and achieve penetration depths required to meet current consensus definitions of clean margins. Compared to routine histological analysis requiring 3–5 days, OCT has the potential to be used for rapid (within several minutes) intraoperative evaluation of tumor boundaries and negative surgical margins.

Earlier studies demonstrated that quantitative evaluation of OCT images with the determination of attenuation coefficients provides substantially improved contrast over its qualitative analysis or description, delineating nuanced features within breast cancers and potentially improving resection margin assessment [30,42]. However, variations in the attenuation coefficient calculated based on conventional structural OCT introduced by different breast tissue types within dense benign and tumorous tissue could contribute to the overlap in the attenuation coefficient values between these groups, which complicates their differentiation. CP OCT technology detects polarization-dependent changes and, therefore, represents a promising tool for the assessment of the state of connective tissue in breast cancer and its differentiation from areas of tumor cell clusters based on the registration of cross-polarization backscattering of the OCT signal [29,38]. In addition, in this study, a more recent method of measuring the attenuation coefficient, known as the depth-resolved approach [42,43,46], was used, while in other studies on the evaluation of attenuation coefficient in breast tissue linear fitting method was used [30]. The advantages of the depth-resolved approach involve avoiding axial resolution deterioration from the fitting range and inaccurate attenuation coefficient measurements due to a poor choice of fitting range. In addition, it is worth noting that averaging across the A-scans could significantly reduce the speckle noise, thus improving the estimation of the attenuation coefficient for the cost of the reduced resolution. In the present study, the trade-off between the accuracy of the attenuation coefficient estimation and the resolution of the resulting distributions was resolved by applying averaging in the local 3 × 3 × 3 pixel window before the attenuation coefficient estimations.

Overall, in this study, using a depth-resolved approach for attenuation coefficient calculation and building color-coded Att(co) and Att(cross) coefficient maps allowed detailed visualization of breast cancer specimens by providing a much higher contrast between different breast tissues within various breast cancer subtypes compared to conventional structural OCT images and linear fitting for attenuation coefficient calculation. In our study, the construction of Att(cross) coefficient maps allowed us not only to sharpen the contrast of breast lesions but also to improve correspondence to histology data. In 2014, the American Society of Surgical Oncology stated that tumor (invasive cancer or DCIS) not touching the ink at the specimen edge is acceptable to prevent local recurrence [55,56]. Based on the results of two separate meta-analyses, the current consensus states that the negative margin for invasive breast cancer is the absence of tumor cells in the inked edge of the resected specimen, and for DCIS, the appropriate margin is greater than 2 mm. The evaluation of the Att(cross) coefficient allowed for the differentiation of more precisely five breast tissue types (adipose tissue, non-tumorous fibrous connective tissue, hyalinized tumor stroma, high-density tumor cells, and low-density tumor cells in fibrotic tumor stroma) and to achieve a more contrasted border between tumorous and non-tumorous tissue as well as a correlation between the morphological structure of the tumor and the scattering/polarization properties of the tissue. Specifically, based on the calculation of attenuation coefficients, it was shown that attenuation imaging in cross-channel facilitates the identification of variations in tumor cell density from the surrounding tumor stroma with a different state. This is likely because the polarization mode used here is connective tissue-targeted; this makes it possible to assess the features of the state of the connective tissue of the breast, visualize changes in the tumor stroma and clearly separate it from clusters of tumor cells.

The observations reported in Figure 3, Figure 4, Figure 5 and Figure 6 regarding the relationship between breast tissue morphology and attenuation patterns showed a wide range of breast tissue types appearing as different patterns on the attenuation coefficient maps. Figure 7 summarizes the findings of this study and demonstrates statistically significant differences between the various breast tissue types. As a result, this made it possible not only to distinguish large clusters of tumor cells from the stromal tissue but, with high statistical significance (*p* < 0.0001), to separate cells with low density in the fibrotic tumor stroma from the surrounding non-tumorous fibrous connective tissue. The Att(co) coefficient box plots in Figure 7A show a large overlap between non-tumorous fibrous tissue and low-density tumor cells in fibrotic tumor stroma and hyalinized tumor stroma. Hypothetically, this is due to low-density cells and dense connective tissue having a similar scattering level. At the same time, the calculation of the Att(cross) coefficient (Figure 7B), detecting the polarization properties of the tissue, showed higher statistically significant differences between all the studied breast tissues (*p* < 0.0001). Measurements of tumor cell density may assist intra-operationally by ensuring a clean margin of the resection during BCS and determining the pathological response of cancer to neoadjuvant chemotherapy.

In addition, color-coded attenuation coefficient maps of these specimens demonstrated that various degenerative changes of the tumor stroma (fibrosis or hyalinosis) could also be differentiated. The presence of hyalinosis of the tumor stroma indicates its secondary deeper (degenerative) changes [52]. During the study, it was found that areas of hyalinosis of collagen fibers are characterized by a decrease in Att(co) and an increase in Att(cross) coefficient compared to the fibrous tissue. Earlier studies demonstrated that denser and highly scattering malignant tumor tissue could be difficult to differentiate from normal connective tissue due to similar optical refractive index and scattering intensity [57]. In this regard, the additional use of median values of the Att(cross) coefficient can be useful in cases where predominated colors on the optical maps reflect the values of the coefficients between adjacent tissue types (low-density tumor cells/fibrosis or hyalinized stroma). Previously, when calculating the traditional attenuation coefficient, such significant distinctions of breast tissue types were not shown.

This is the first study where local attenuation coefficients of different breast tissues were identified in different molecular and morphologic breast cancer subtypes. In the case of high-grade breast cancer subtypes associated with poor treatment prognosis, the cancer zones demonstrate decreased attenuation coefficient with a fairly homogeneous spatial distribution. In these histological data, this corresponds to a decrease in the content of the stromal component and the predominance of regions of tumor cells. In the case of low-grade breast cancer subtypes and benign fibroadenoma associated with good treatment prognosis, the tumor cell areas demonstrate an increased attenuation coefficient with a fairly heterogeneous spatial distribution. In these histological data, this corresponds to predominance in the content of the stromal component and a small number of regions in tumor cells or ducts/lobules, each of which may give rise to a different Att(cross) coefficient. It is important to take into account that in low-grade invasive carcinoma of no special type on the border with normal tissue, the density of tumor cells can be considerably reduced; also, not only adipose tissue but also fibrous tissue with areas of hyalinosis can constitute the border. Therefore, characteristic values of the attenuation coefficient reflect the changes in the morphological structure of different breast cancers and can be used as a potential biomarker to predict the molecular subtype of breast cancer. This can facilitate more precise breast cancer border identification, as well as assessment of the resection margin.

For the first time, we evaluated the diagnostic ability of Att(co) and Att(cross) coefficients to differentiate tumorous from non-tumorous breast tissue. In this study, optimal threshold values of both attenuation coefficients for detecting tumor cell areas and hyalinized tumor stroma among the surrounding non-tumorous adipose or connective tissues were determined. Overall, for the studied breast cancer, diagnostic parameters for the Art(cross) coefficient demonstrated better values compared to Att(co) coefficient. The CP OCT-based Att(cross) coefficient was able to detect areas of tumor cells with 99% diagnostic accuracy, 99% sensitivity, and 99% specificity in the surrounding non-tumorous fibrous connective tissue (see Figure 8, red line). Att(co) coefficient was able to detect areas of tumor cells with 99% diagnostic accuracy, 99% sensitivity, and 99% specificity in the surrounding adipose tissue (see Figure 8, blue line). In addition, it was demonstrated that Att(cross) coefficient allows for better differentiation of non-tumorous fibrous connective tissue from hyalinized tumor stroma (91%) (see Figure 8, green line). The use of quantitative processing of OCT data with threshold values of attenuation coefficients makes it possible to objectify the data and increase the diagnostic accuracy of this method, and to the best of our knowledge, we are the first to demonstrate this result. High diagnostic accuracy of the Att(cross) coefficient, in comparison with Att(co) coefficient, allows tumor cells and tumor stroma to be better visualized in the surrounding non-tumorous fibrous connective tissue for achieving clear resection margin during BCS and evaluation of the efficacy of neoadjuvant tumor therapy. Thus, we demonstrated the high diagnostic potential of using Att(co) and Att(cross) coefficients for detecting tumor cell areas and minimizing the risk of recurrence and re-resection. Other researchers have demonstrated the dynamics of the decrease or increase in attenuation coefficient values in different breast tissues, which are arbitrarily based on the authors’ clinical experience [30].

Along with the promising results, we would like to highlight several limitations of our study. On the one hand, we need to mention the limitations of the OCT method, such as the low penetration depth of the probing light inside the tissue, which is 1.5 mm. This aspect also includes the small size of selected OCT images and, consequently, the small volume of tissue scanning. In addition, the areas of coagulation and hemorrhage may also lead to changes in the nature of the received OCT signal and the corresponding optical attenuation, which is important in the case of the *in vivo* application during breast cancer surgery. The richness of the extracted information from OCT examination may be comparable with histology, although the resolution of OCT-scans ~10–15 µm is lower than in microscopic histological studies. In addition, co-registration of the histology may be difficult in some cases because of tissue deformation and shrinkage after the formalin fixation and processing. This complicates automated correlation and one-to-one mapping between tissue type and optical attenuation. In addition, in some cases, when high-density tumor cells are present at the tumor border, in the color-coded attenuation coefficient maps, it was difficult to identify the border of adipose tissue because they had similar values. In such cases, it is preferable to use structural log-scale OCT images or elastography OCT-based imaging, as was demonstrated earlier [18,28,29].

In the future, once it becomes possible to acquire a large number of samples, we are planning to apply machine learning and deep neural networks for differentiating breast tissue types and surgical margins assessment using attenuation coefficient values based on volumetric CP OCT data. This will enable building a classifier for automated identification of the various breast tissue type and performing automatic cancer detection by combining the attenuation metrics with additional intensity parameters information on the tissues. Furthermore, studies are needed for a comprehensive evaluation in the intra-operative setting.

To summarize, the CP OCT method with quantitative measuring of the attenuation coefficients is a promising tool for intraoperative human breast tissue differentiation during surgical resection of breast cancer or for performing a targeted histological biopsy for the assessment of the resection margin as well as evaluation of the efficiency of neoadjuvant therapy. Furthermore, we believe that the results reported here represent a baseline in the use of this technique and are a first step towards establishing its use in a clinical setting. Current efforts are underway for the construction and implementation of a handheld CP OCT probe for in vivo imaging during BCS and real-time resection margins assessment.

## 5. Conclusions

In this study, we have shown qualitatively and quantitatively that color-coded attenuation coefficient maps based on CP OCT imaging may enable better visualization of detailed features in different subtypes of breast cancer. We showed that mapping of the attenuation coefficient in co- and cross-polarization channels using the depth-resolved approach presented here shows great promise for automated classification of different tissue types in human breast tissue (adipose tissue, non-tumorous fibrous connective tissue, hyalinized tumor stroma, high-density tumor cells and low-density tumor cells in fibrotic tumor stroma). Att(cross) coefficient is more suitable for differentiation of tumor cell areas of different densities from non-tumorous fibrous tissue: diagnostic accuracy was 91–99%, sensitivity—96–98%, and specificity—87–99%. Att(co) coefficient is more suitable for the differentiation of tumor cell areas from adipose tissue: diagnostic accuracy was 83%, sensitivity—84%, and specificity—84%. We believe this methodology provides an important improvement to conventional OCT imaging of different breast tissue type morphology and is a step towards in vivo assessment of the surgical margin of breast cancer resection using attenuation coefficients based on volumetric CP OCT data.

## Figures and Tables

**Table 1:** Summary of diagnostic performances of Att(co) and Att(cross) coefficients for detection of tumor tissue.

	AUC (95% CI)	Sensitivity (95% CI)	Specificity (95% CI)	Diagnostic Accuracy (95% CI)	The Optimal Cutoff (Pth), Yielding Maximal Sum of Sensitivity and Specificity
	Attenuation (co) coefficient				
Tumor cell areas vs. Adipose tissue	0.91 (0.85; 0.96)	84% (74%; 91%)	84% (68%; 93%)	83% (78%; 86%)	>4.2 mm^−1^
Tumor cell areas vs. Non-tumorous connective tissue	0.83 (0.75; 0.90)	65% (53%; 75%)	91% (77%; 97%)	78% (71%; 90%)	<4.8 mm^−1^
Hyalinized tumor stroma vs. Non-tumorous connective tissue	0.56 (0.44; 0.68)	60% (39%; 78%)	56% (45%; 68%)	58% (45%; 70%)	<5.2 mm^−1^
	Attenuation (cross) coefficient				
Tumor cell areas vs. Adipose tissue	0.71 (0.61; 0.82)	68% (56%; 78%)	66% (50%; 80%)	67% (67%; 88%)	>1.1 mm^−1^
Tumor cell areas vs. Non-tumorous connective tissue	0.99 (0.99; 1.0)	98% (91%; 99%)	99% (91%; 1.0%)	99% (98%; 1.0%)	<3.1 mm^−1^
Hyalinized tumor stroma vs. Non-tumorous connective tissue	0.97 (0.93; 1.0)	96% (82%; 99%)	87% (74%; 94%)	91% (84%; 97%)	>4.3 mm^−1^

AUC—area under ROC curve; Cl—confidence interval; Pth—threshold value.

## Data Availability

The data presented in this study are available on request from the corresponding author.
